# Design and Fabrication of a Large-Stroke Deformable Mirror Using a Gear-Shape Ionic-Conductive Polymer Metal Composite

**DOI:** 10.3390/s120811100

**Published:** 2012-08-09

**Authors:** Hsiang-Chun Wei, Guo-Dung John Su

**Affiliations:** Graduate Institute of Photonics and Optoelectronics, National Taiwan University, No. 1, Roosevelt Road, Section 4, Taipei 10617, Taiwan; E-Mail: d96941017@ntu.edu.tw

**Keywords:** Nafion^®^, IPMC, deformable mirror, FEM, grey box model

## Abstract

Conventional camera modules with image sensors manipulate the focus or zoom by moving lenses. Although motors, such as voice-coil motors, can move the lens sets precisely, large volume, high power consumption, and long moving time are critical issues for motor-type camera modules. A deformable mirror (DM) provides a good opportunity to improve these issues. The DM is a reflective type optical component which can alter the optical power to focus the lights on the two dimensional optical image sensors. It can make the camera system operate rapidly. Ionic polymer metal composite (IPMC) is a promising electro-actuated polymer material that can be used in micromachining devices because of its large deformation with low actuation voltage. We developed a convenient simulation model based on Young's modulus and Poisson's ratio. We divided an ion exchange polymer, also known as Nafion^®^, into two virtual layers in the simulation model: one was expansive and the other was contractive, caused by opposite constant surface forces on each surface of the elements. Therefore, the deformation for different IPMC shapes can be described more easily. A standard experiment of voltage *vs.* tip displacement was used to verify the proposed modeling. Finally, a gear shaped IPMC actuator was designed and tested. Optical power of the IPMC deformable mirror is experimentally demonstrated to be 17 diopters with two volts. The needed voltage was about two orders lower than conventional silicon deformable mirrors and about one order lower than the liquid lens.

## 1. Introduction

Because of the extreme competition within the digital camera industry, camera modules with good image quality, optical power varying, and compact size are a critical issue. A typical focusing or zooming camera module varies the optical power, which also means focal length or focal power, by moving the interior lenses with the use of motors. The space occupied by the actuating motor and the reserved space for the moving lens increase the volume of this camera image module. There are two alternative solutions for varying the optical power of a camera module without moving any lens. One solution is the refractive-type design. The liquid lens announced by Varioptic^®^ [1] adopted two immiscible liquids that can deform the shape of the bi-liquid interface by an electro-wetting effect. By changing the curvature of the bi-liquid interface in the lens, this camera module will change the optical power. Liquid crystal lenses [2] belong to the refractive-type as well. The focal length can be varied from the value *f_e_* for an extraordinary ray to *f_o_* for an ordinary ray by applying an electric field across the lens-cell. The other proposed solution is a reflective-type design, which adopts a Micro-electromechanical Systems (MEMS) deformable mirror to vary the optical power of the camera module. Wick *et al.* [3] demonstrated that active optical elements with variable focal length mirrors can be used to eliminate mechanical motion by motors in zoom lens systems. Changing the surface of the deformable mirror manipulates the optical power of the module.

A deformable mirror (DM) is a critical component that can vary focal length by changing the surface deformation. Within an optical system, auto focus and optical zoom can be achieved by using DMs. This has advantages over other refractive type components. Liquid lenses encounter gravity, dispersion, shaking and temperature issues. Liquid crystal lenses suffer from dispersion, temperature, polarizers, and additional AC voltage application issues. A DM has the advantages of small size compared to voice coil motors, freedom from dispersion and resistance to vibration. Silicon and polymer based MEMS deformable mirrors have been demonstrated [4,5] successfully. However, the high actuation voltage and unidirectional focal length variation limited their applications.

**Figure 1.**
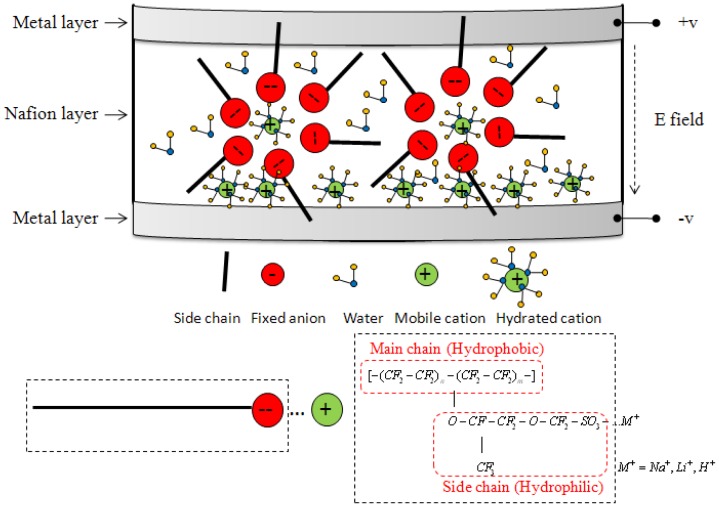
Schematics of the electro-osmotic migration of hydrated counter-ions within the IPMC network. Black bars and red balls stand for hydrophilic side chains and hydrophobic main chains, respectively.

Ionic polymer-metal composite (IPMC) is a promising alternative material for use in fabricating MEMS-based DMs because of its ability to exhibit large bidirectional actuation with low applied voltage. Figure 1 shows the schematics of the electro-osmotic migration of hydrated counter-ions within the IPMC network. It is a sandwich structure with a layer of Nafion^®^ inside and two layers of metal outside as the electrodes. The chemical formula of Nafion^®^ can be separated into two chains. The hydrophobic main chain forms the backbones to determine the mechanical strength, which is the black bar. The hydrophilic side chain terminated by ionic groups, such as SO_3_^−^ for cation exchange, is the red ball noted fixed anion. The working principle of IPMC actuation is that when an electric field is applied, hydrated cations move through the cluster networks which are formed by the chains towards the cathode so that the volume expands near the cathode side and contracts near the anode side. As a results the IPMC bends toward the anode. According to the actuation mechanism, the real internal stress inside Nafion^®^ should be symmetric and linear distributed along its thickness [6], which is positive in one layer and is negative in another layer of Nafion^®^. Traditionally, the IPMC in cantilever beam shape can only generate bending motions, but actuators with the capability of complex deformation are highly desirable in many applications. Pugal *et al.* [7] presented an electrode patterned IPMC with a twist motion for bionics applications. In recent works [8], we proposed a three-deformational gray box model based on the finite element method (FEM). According to this model, the deformation of IPMC in arbitrary shapes which were confined with different boundaries can be predicted more easily. Therefore, arbitrary deformation can be achieved and pre-designed. In this work, a gear-shape IPMC was designed and demonstrated to deform like a rotationally symmetric, or aspheric, surface. The experimental results agreed well with the simulation data which will be discussed in the latter sections. However, IPMC is known for practical challenges, such as reliability in dry air and the back-relaxation phenomenon. This is mainly due to the actuation mechanism which depends on the movement of hydrated cations inside it. Therefore, the effects of water content on the actuation performance of ionic polymer–metal composites are quite important [9]. Thereafter, encapsulation processes applied to IPMCs are nowadays a critical subject to their practical use. PDMS and parylene are adopted as encapsulants to improve the performance of IPMC [10]. The PDMS layer which was adopted in this research can not only smooth the surface but also be treated as an encapsulation process to improve the performance. There are many applications using IPMC as an actuator. We believe that this paper is the first effort to implement IPMC in a three-dimension deformable mirror area.

## 2. Three-Dimensional Gray-Box Deformation Model

**Figure 2.**
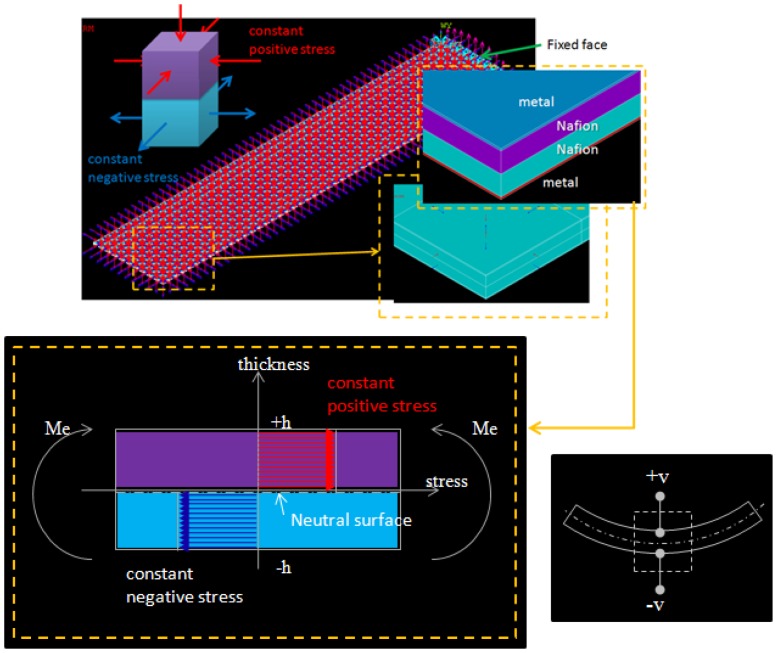
The structure and ANSYS^®^ element model of a cantilever beam shaped IPMC with the actuation mechanisms. Red and blue arrows stand for compressive and tensile stress. This configuration shows IPMC bent toward the anode.

According to the actuation mechanism, the real internal stress inside Nafion^®^ should be symmetric and linearly distributed along the thickness, which is positive in one layer and is negative in another layer of Nafion^®^. However, this real model is not easy to apply to arbitrary shapes and boundary confinements. Therefore, we developed a gray-box FEM model to simplify the simulation and prediction. The FEM model was used to perform a quick prediction for three-dimensional deformation in arbitrary shapes. Since the deformable mirror is used as a light reflector, we care about shape, instead of force or current draw. An IPMC was divided into four layers along the thickness by different materials and opposite stresses. There were two layers of metallic electrodes outside the IPMC, a layer of Nafion^®^ with constant compressive surface stress, and a layer of Nafion^®^ with constant tensile surface stress inside the IPMC. The total thickness was 2(*h_Nafion_* + *h_metal_*) and there was an effective bending moment, noted as Me, caused by the internal stresses. There was a neutral surface with no stress between the two Nafion^®^ layers. Figure 2 shows the structure and element model which were designed by ANSYS^®^ for a cantilever beam shaped IPMC. All the stresses were applied normally to the element surfaces. The stresses were positive in the upper layer and negative in the lower layer of Nafion^®^. Red arrows and blue arrows stand for the compressive and tensile stresses, respectively. The element type which was chosen in ANSYS^®^ was SOLID 45, which is used for the three-dimensional modeling of solid structures. The element is defined by eight nodes each having three degrees of freedom at each node. The physical properties were follows: Young's modulus of platinum and Nafion^®^ were 168 GPa and 275 MPa, respectively. Poisson's ratio of platinum and Nafion^®^ were 0.38 and 0.487, respectively. All the physical parameters are listed in Table 1.

**Figure 3.**
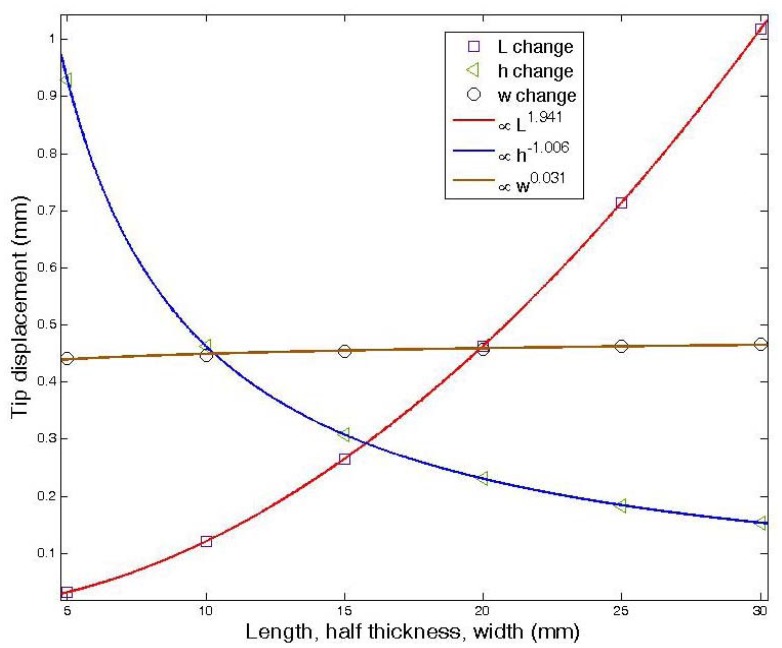
FEM simulation which varied the length (*L*) and width (*w*) of IPMC and the half thickness (*h*) of Nafion^®^ with constant surface force (*P* = 100 Pa).

**Figure 4.**
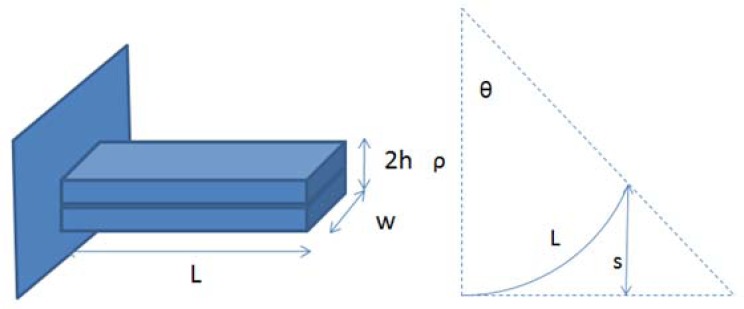
The schematic diagram of the geometry parameters of a cantilever beam shaped IPMC and the tip displacement.

From the simulation of the FEM by varying the length and width of IPMC and the half thickness of Nafion^®^, as shown in Figure 3, with surface stress equal to 100 Pa, we could derive the relation of surface stress to tip displacement. According to this Figure, we try to determine the effects of the geometry on the tip displacement. This result leads to Equation (1):

(1) $$s_{\text{ansys}}\propto L^{2}h^{-1}\cdot P$$

where *S_ansys_* is the simulated max tip displacement, *L* is the length, *h* is the half thickness of Nafion^®^, and *P* is the surface stress on each element. We also simplified the Nasser's analytical solution [11],

(2) $$Me\dot{=}k_{0}k_{e}\phi_{0}ahw$$

from the relations:

(3) $$M_{k}=\frac{1}{\rho }EI[12]$$

(4) $$\text{and}\;I=\frac{wh}{6}(4h^{2}+w^{2})≅\frac{2wh^{3}}{3}[12]$$

(5) $$s=\rho (1-\cos\frac{L}{\rho })$$

(6) $$\text{to}\;s=\frac{EI}{m_{e}\phi_{0}hw}2\sin^{2}\left(\frac{m_{e}\phi_{0}hwL}{2EI}\right)$$

where *k_0_*, *k_e_* and α are material constant, *m_0_* = *k_0_k_e_α*, *w* is width, *M_k_* and *M_e_* are the bending moment, *ρ* is radius of curvature, *E* is Young's modulus, *I* is moment of inertia, and *s* is max tip displacement as shown in Figure 4. From Equations (2) and (3), we let $M_{k}=M_{e},\text{so}\;\rho =\overset{EI}{\;}/\underset{m_{e}\phi_{0}hw}{\;}$. Then after taking it into Equation (5) and simplifying it by the triangle formula, Equation (6) can be finally derived. Equation (6) is approximately linear with low applied voltage. Voltages of less than 10 V make this approximation valid. The voltage is limited by the electrolysis of water, so we didn't apply any voltage larger than 5 V:

(7) $$s≅\frac{m_{e}\phi_{0}hwL^{2}}{EI}=\frac{3}{2}\frac{m_{e}\phi_{0}L^{2}}{Eh^{2}}\propto \frac{L^{2}}{h^{2}}\phi_{0}$$

Then, comparing the simulated Equation (1) with the simplified Nasser's analytical solution of Equation (7) [11], making *s_ansys_* equal to *s*, we could derive the relationship of the surface stress and applied voltage, which was:

(8) $$P=\overset{c_{1}}{\;}/\underset{h^{\cdot \phi_{0}}}{\;}$$

where *P* is the surface stress on each element, *φ*_0_ is applied voltage, *h* is the half thickness of Nafion^®^, and *C_1_* is constant which depends on the characteristic of Nafion^®^. For different process conditions, such as temperature, concentration of metal salt solution, and cation concentration, the characteristics of Nafion^®^ are different and therefore *C_1_* is different. From Equation (8), it is revealed that the relation between surface stress and applied voltage just depends on the thickness. Meanwhile, according to Equation (8), once the parameter *C_1_* is determined experimentally, we can simulate the deformation profile of any arbitrary shape IPMC with the given voltage. We used the experimental result of voltage *vs.* tip displacement to determine the constant, *C_1_*. Thus, the constant was approximately 60,000 in this work. The experimental results will be discussed latter.

## 3. Fabrication Process of a Gear Shaped IPMC

**Figure 5.**
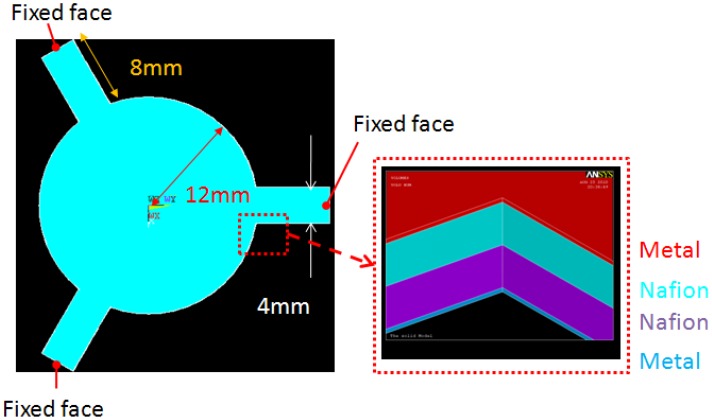
The structure model in ANSYS^®^ of a gear shaped IPMC design.

**Figure 6.**
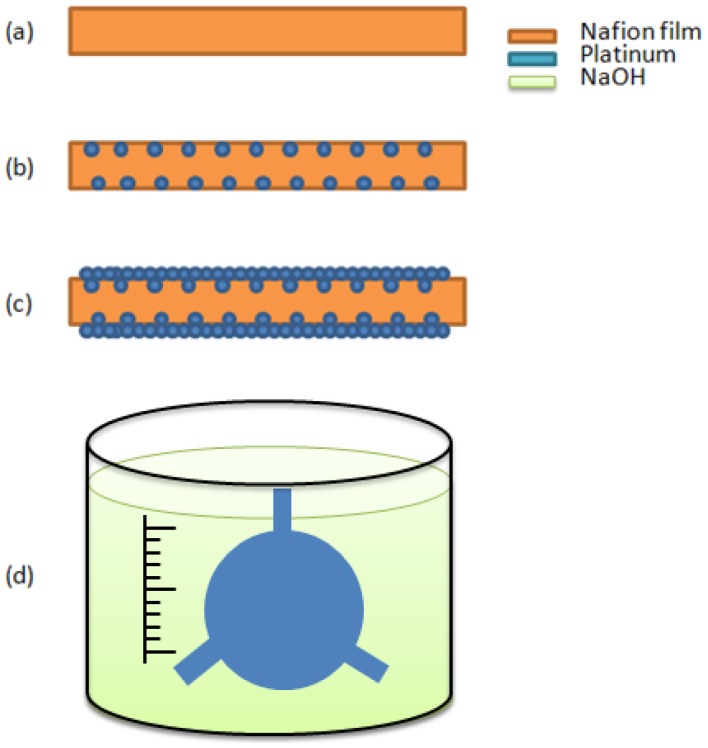
The IPMC process flow. (**a**) ion exchange polymer formation and pre-process; (**b**) initial compositing; (**c**) surface electrode growing; (**d**) shape cutting and immersing in the salt solution.

A new deformable mirror with a gear-shaped IPMC design was presented in this paper. The designed model is shown in Figure 5. There were three fixed arms and a free mirror at the center. The mirror was 12 mm in radius, the length and the width of the fixed arm was 8 mm and 4 mm, and the thickness of metal and half thickness of Nafion^®^ were 10 μm and 100 μm, respectively. The size was chosen to achieve the desired deformation for optical applications. There are four major steps to make IPMC actuators: (a) ion exchange polymer (often called ionomer) formation and pre-processing; (b) initial compositing; (c) surface electrode growing; (d) shape cutting. Figure 6 shows the fabrication process of the gear-shape IPMC deformable mirror. The effective recipe (Sections 3.1–3.3, [13]) used to manufacture the IPMC materials was based on electroless plating with some minor revisions.

### 3.1. Ion Exchange Polymer Formation and Pre-Process

The step shown in Figure 6(a) involved making a sheet of ion exchange polymer membrane, which could be found in commercial Nafion^®^ film (DuPont, Wilmington, DE, USA) or fabricated using Nafion^®^ liquid solution. We adopted commercial Nafion^®^ 117 film. The thickness was approximately 200 μm. The pre-procesings included three steps. The first was surface roughening on both side of membrane by using 1,000 Cw sandpaper. The roughening increases the effective area of Nafion^®^ available to hold the metal layer. For cleaning the surface debris and dirt, an ultrasonic cleaner (Model 2510, Branson, Danbury, CT, USA) was used for 10 min to clean each side of the Nafion^®^ membrane. The second was acid cleaning with boiling 2.4 N aqueous HCl followed by boiling DI water 100 °C for 45 min to remove the impurities and to activate the membrane with H^+^ ions. The third was soaking in the salt solution. The salt solution was 0.2% Pt(NH_3_)_4_HCl, which would diffuse platinum-containing positive ions into the inner surface a few micrometers deep in the ion exchange polymer via an ion exchange process.

### 3.2. Initial Compositing

In Figure 6(b), the initial compositing processes, sparsely distributed metallic particles were buried in the inner surface of the ion exchange polymer by an electroless-plating method to form a metallic particle foundation layer for step (c) in Figure 6(c). The primary reaction was:

(9) $$\begin{array}{l} NaBH_{4}+4{\left[Pt(NH_{3})\right]}^{2+}+8OH^{-}\\ \Rightarrow 4Pt^{0}+16NH_{3}+NaBO_{2}+6H_{2}O \end{array}$$

where 2 mL NaBH_4_ 5 wt % was added every 30 min for four hours while gradually increasing the temperature from 40 °C to 60 °C. The reaction was carried out in a water bath. The highly dispersed metallic particulate layer was buried a few micrometers deep under the interface boundaries. Typically the reducing agent is NaBH_4_. In the last part of step (b) in Figure 6, the membrane with highly dispersed metallic particulate layer was rinsed with DI (deionized) water to remove the residual metallic ions and then stored in a 0.5% solution of HCl for 8 hours to increase the H^+^ ion concentration.

### 3.3. Surface Electrode Growing

In Figure 6(c), the surface electrode grows the metallic particles smoothly on both side surfaces of the IPMC using a stronger reducing agent. A solution of 300 mL DI water with 0.2 g Pt(NH_3_)_4_HCl and 0.6 mL NH_4_OH 25% was prepared in water bath at 40 °C. The stronger reducing agent used included Hydroxylamine hydrochloride (H_2_NOH·HCl) 5 wt % and H_2_NNOH_2_·H_2_O 20%. At first, the residual HCl of the membrane was removed by rinsing in DI water. The reducing agent was added, 3 mL and 1.5 mL, rspectively every 30 min for four hours while gradually increasing the temperature from 40 °C to 60 °C. The surface resistivity becomes greatly reduced.

### 3.4. Shape Cutting

Finally, the IPMC sheet was completed and can be cut into a gear shape by using a laser cutting machine (Figure 6(d)). We then soaked it in an appropriate salt solution, such as NaOH, to add counter-ions for the subsequent measurements.

## 4. Experimental Results

### 4.1. Cantilever Beam Shape IPMC

**Figure 7.**
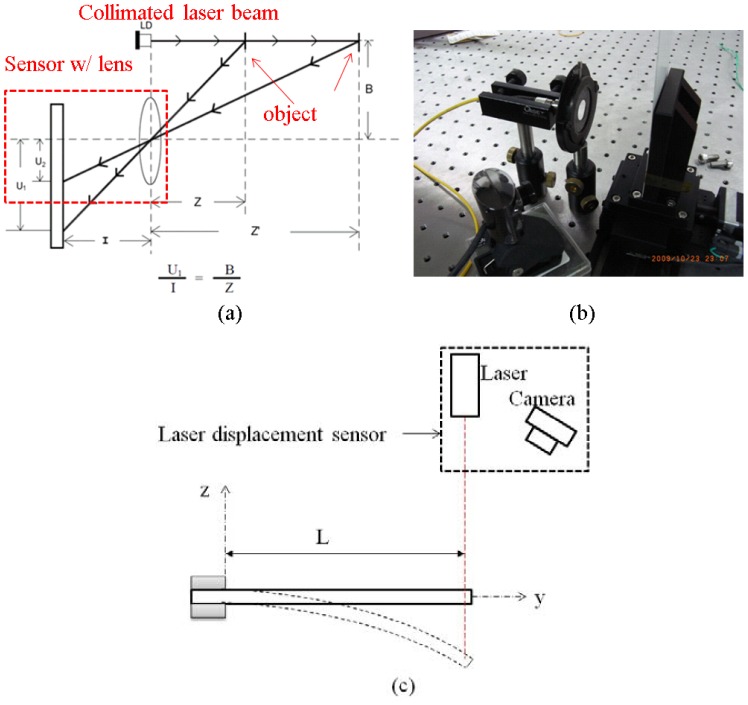
The laser displacement based on triangulation measurement method. (**a**) the triangulation measurement method; (**b**) the real components of the laser displacement sensor; (**c**) the schematic measurement setup for max tip displacement of cantilever beam shape IPMC.

**Figure 8.**
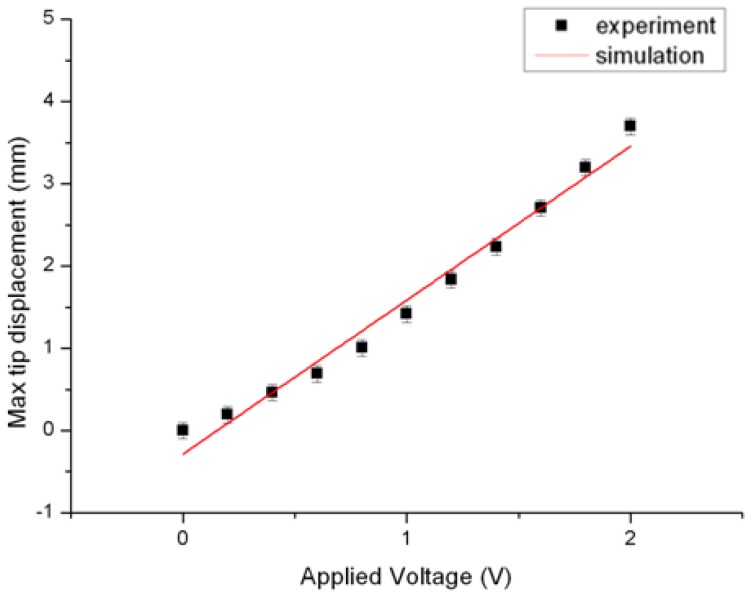
The agreement of experimental and simulation results for max tip displacement *vs.* applied voltage.

In the cantilever beam experiment, the length was 20 mm, the width was 5 mm, and the thickness of platinum and the half thickness of Nafion^®^ were 10 μm and 100 μm, respectively. In this work, the constant *C_1_* of IPMC was 60,000. The max tip displacement was measured by a homemade laser displacement sensor as shown in Figure 7. The laser displacement sensor setup in Figure 7(a) was based on a triangulation measurement method. It included a collimated laser beam source, a sensor with lens, and a motorized linear stage (KS112, Suruga, Shizuoka, Japan). The motorized linear stage was used for system calibration. Meanwhile, the displacement of a spot image on the two-dimensional sensor was transferred to the displacement of the object. Figure 7(b) shows the real components of the laser displacement sensor. The resolution of the system was approximately 0.1 mm. Figure 7(c) shows the schematic measurement setup for max tip displacement of cantilever-beam-shaped IPMC. Figure 8 shows the good agreement of experimental data and simulation result.

### 4.2. Gear Shaped IPMC

**Figure 9.**
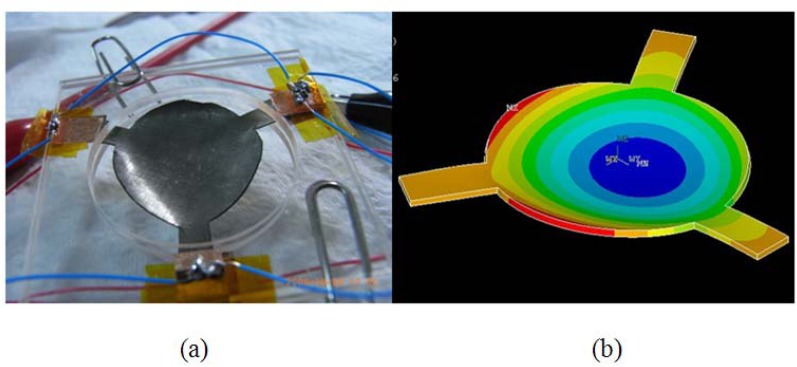
(**a**) Picture of the experimental demonstration of the new deformable mirror; (**b**) The deformation simulated by ANSYS^®^.

**Figure 10.**
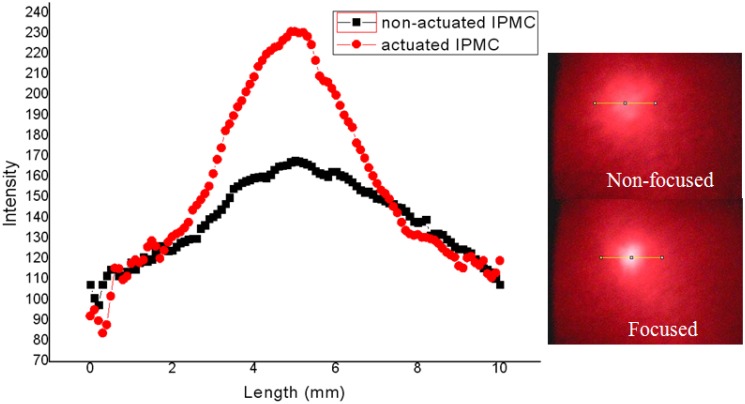
The intensity profile of the laser beam spot with non-actuated IPMC (non-focused) and actuated IPMC (focused).

**Figure 11.**
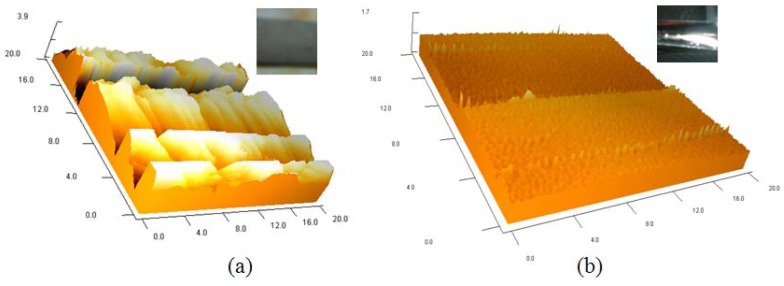
The surface profile of IPMC using an atomic force microscope (AFM). (**a**) the non-improved surface (R_rms_ = 1.29 μm); (**b**) the improved surface (R_rms_ = 0.036 μm).

Figure 9(a) is the picture of the fabricated IPMC deformable mirror. The central displacement was approximately 0.6 mm and the corresponding optical power was 17 diopters (m^−1^) under two volts of applied voltage. Figure 9(b) shows the deformation profile which was simulated by ANSYS^®^. The experimental result of a reflected laser spot by flat and curved IPMC DM is shown in Figure 10. The laser spot was focused on a point approximately 6 cm from the DM. The corresponding optical power was approximately 17 diopters. Because of the diffused metal particles caused by surface roughening in step (b) in Figure 6 of the fabrication process, the laser spot was somewhat scattered. Comparing with a flat IPMC mirror, the actuated IPMC mirror focused 50% more energy in the 2 mm diameter. In other words, the collimated light was focused successfully. Figure 10 illustrates the intensity profile of the laser spot reflected by the non-actuated IPMC (non-focused) and the actuated IPMC (focused). To overcome this phenomenon, we spun a layer of polydimethylsiloxane (PDMS) as a buffer layer to make the surface smoother. Meanwhile, a layer of 500 nm metal reflector was deposited by an e-beam evaporator. The comparison of improved surface roughness is shown in Figure 11 by using an atomic force microscope (AFM; OBJ-204C, ITRI, Hsinchu, Taiwan). Figure 11(a) shows the non-improved surface with scars caused by sandpaper. Figure 11(b) shows the improved surface. The root-mean-square roughness was improved from 1.29 μm to 0.036 μm. According to previous work by Bar-Cohen *et al.*, [14], encapsulation techniques were also investigated to successfully preserve the moisture content when the voltage level is below two volts. Meanwhile, Nemat-Nasser [15] presented results using ethylene glycol as solvent. They found out that IPMC with ethylene glycol has greater solvent uptake. It can be subjected to higher voltages without electrolysis. Compared to water, it can be actuated in open air for long time periods.

All these experimental results demonstrated that the gear shaped IPMC DM had suitable optical focusing power and needed much lower voltage than other techniques, such as liquid lenses and micromachined electrostatic deformable mirrors. The liquid lens needed 60 V to achieve ±15 diopters and micromachined electrostatic deformable mirrors required 160 V for 20 diopters. The comparison of these techniques is shown in Table 2.

## 5. Conclusions

In this paper, we have built a convenient gray box model based on the finite element method to simulate the deformation of a gear shaped IPMC. The gear shaped IPMC provided a surface pull down motion which was different from the conventional cantilever bending motion. The simulation model considered the mechanical properties of Nafion^®^ and metal, including Young's Modulus and Poisson's ratio. There was a constant which depends on the processing of the IPMC. Therefore, a cantilever beam shaped IPMC should be manufactured and measured first to determine the constant by the experiment of max tip displacement *vs.* applied voltage. The three-dimensional deformation of the IPMC actuator can be predicted much more easily by using the convenient gray box model. Because of the diffused metal particles caused by surface roughening, the laser spot is somewhat scattered. The root-mean-square roughness was improved from 1.29 μm to 0.036 μm by using a PDMS buffer layer. The focused light spot was approximately 6 cm from the DM. The corresponding optical power was 17 diopters with two volts of applied voltage.

## Figures and Tables

**Table 1.** The physical parameters.

	**Young's Modulus**	**Poisson's Ratio**
**Platinum**	168 GPa	0.38
**Nafion^®^**	275 MPa	0.487

**Table 2.** Comparison between different technologies.

	**Liquid Lens** [1]	**Liquid Crystal Lens** [2]	**MEMS Deformable Mirror** [5]	**IPMC Deformable Mirror (exp)**
**Color dispersion**	Yes	Yes	No	No
**Applied voltage**	60 V	5 V	160 V	2 V (lowest)
**Max optical power**	±15 diopter	±5 diopter	20 diopter	±17 diopter
**Other issues**	Gravity problems Temperature	Additional AC circuit; Temperature Polarizer needed	High applied voltage	Complicated control for accurate displacement
